# Raman Spectroscopy Technique: A Non-Invasive Tool in Celiac Disease Diagnosis

**DOI:** 10.3390/diagnostics11071277

**Published:** 2021-07-16

**Authors:** Giuseppe Acri, Claudio Romano, Stefano Costa, Salvatore Pellegrino, Barbara Testagrossa

**Affiliations:** 1 Dipartimento di Scienze Biomediche, Odontoiatriche, e delle Immagini Morfologiche e Funzionali, Università degli Studi di Messina, 98125 Messina, Italy; 2 Unità Operativa Semplice Dipartimentale Gastroenterologia Pediatrica e Fibrosi Cistica, Azienda, Ospedaliera Universitaria Policlinico G. Martino, Via Consolare Valeria, 98125 Messina, Italy; claudio.romano@unime.it (C.R.); stefano.costa@polime.it (S.C.); salvatore.pellegrino@polime.it (S.P.)

**Keywords:** Celiac disease (CD), Raman spectroscopy, non-invasive methodology, ROC curves

## Abstract

Celiac disease (CD) is diagnosed by a combination of specific serology and typical duodenal lesions. The histological confirmation of CD, mandatory in the majority of patients with suspected CD, is based on invasive and poorly tolerated procedures, such as upper gastrointestinal endoscopy. In this study we propose an alternative and non-invasive methodology able to confirm the diagnosis of CD based on the analysis of serum samples using the Raman spectroscopy technique. Three different bands centered at 1650, 1450 and 1003 cm^−1^ have been considered and the A_1450_/A_1003_ and A_1650_/A_1003_ ratios have been computed to discriminate between CD and non-CD subjects. The reliability of the methodology was validated by statistical analysis using receiver operating characteristic (ROC) curves. The Youden index was also determined to obtain optimal cut-off points. The obtained results highlighted that the proposed methodology was able to distinguish between CD and non-CD subjects with 98% accuracy. The optimal cut-off points revealed, for both the A_1450_/A_1003_ and A_1650_/A_1003_ ratios, high values of sensitivity and specificity (>95.0% and >92.0% respectively), confirming that Raman spectroscopy may be considered a valid alternative to duodenal biopsy and demonstrates spectral changes in the secondary structures of the protein network.

## 1. Introduction

Celiac disease (CD) is an immuno-mediated systemic disorder due to ingestion of gluten proteins of wheat, barley, and rye in genetically susceptible individuals carrying the HLA-DQ2 and/or -DQ8 alleles [1]. CD is characterized by typical duodenal mucosal lesions associated with the presence of CD-specific autoantibodies against tissue-transglutaminase type 2 (tTG) and endomysium (EMA). The prevalence of CD in Europe and North America is about 1%, with higher rates in first-degree relatives of CD patients and individuals with associated disorders such as type 1 diabetes mellitus or trisomy 21 [2,3]. A combination of CD serology testing and duodenal biopsy sampling is required for the diagnosis of CD in adults [4], but not in all children, provided that they meet the criteria suggested by the new ESPGHAN guidelines [5]. These criteria rely on measurement of the concentration of tTG (anti-tTG) IgA antibodies (TGA-IgA). If TGA-IgA is ≥10 times the upper limit of normal (10 × ULN) and the family agrees, the no-biopsy diagnosis may be applied, provided endomysial antibodies (EMA-IgA) will test positive in a second blood sample. HLA DQ2-/DQ8 determination and symptoms are not obligatory criteria. Only if total IgA is low/undetectable, an IgG based test, such as deamidated gliadin peptide antibodies (DGP-IgG), is indicated. In children with positive TGA-IgA <10 × ULN, at least four biopsies from the distal duodenum and at least one from the bulb should be taken; a Marsh 2–3 type histological change confirms the diagnosis. An increased risk of progression towards intestinal cancers has been reported among untreated or undiagnosed celiac patients [6,7]. In this context, an early and accurate diagnosis is crucial to plan adequate therapy. Histologic, serologic, and genetic testing are routinely performed in the clinical evaluation of patients with CD. However, the histological changes of small-bowel mucosal biopsies are still the gold standard to confirm the diagnosis of CD. In the last decade, alternative methodologies, like Raman and infrared spectroscopy, have been proposed as alternative valid tools in clinical diagnosis. Raman spectroscopy is an optical technique based on the inelastic scattering of photons by molecular bonds [8]. The obtained spectrum yields the biochemical information of a specific molecule [9]. Raman Spectroscopy is used to characterize nucleic acids, lipids, carbohydrates and complex biological systems by attributing a specific signature to each sample [10]. It represents an essential methodology in different research fields, such as: chemistry, physics, biology, material sciences and medicine [11,12]. Moreover, Raman spectroscopy has proven to be a versatile tool in clinical diagnostics [13], and has been applied on tissues to detect a variety of diseases ranging from cancer [14,15,16,17,18,19,20,21,22] to infectious diseases [23,24,25,26], neurodegenerative diseases [27] and inflammatory diseases [28,29,30,31,32,33,34,35]. Fornasaro et al. [34] used Raman spectroscopy on tissues to evaluate alterations in the biochemical composition of intestinal tissue associated with CD. Recently [36,37] Raman spectroscopy was used to reveal biochemical differences in the plasma of Crohn’s disease patients and to carry out Celiac disease diagnosis on individual red blood cells, respectively. In this last study [37], chemometric analysis were used in combination with Raman hyperspectroscopy, because insufficient differentiation between average Raman spectra of Celiac disease donors and healthy patients was evident.

The aim of our study concerns an alternative methodology for the diagnosis of CD to simplify procedures and provide a versatile and fast method to distinguish between non-CD and CD patients. It is based on the Raman spectroscopy technique and requires only a serum sample for analysis. The great advantage of the developed methodology consists in its ability to detect an inflammatory disease without any invasive procedure. The reliability of the alternative methodology of diagnosis was evaluated with respect to duodenal biopsy, which represents the gold standard (GS). Statistical analysis was also performed; in particular, the receiver operating characteristic (ROC) curve and Youden index (Y) were computed, and the obtained values were used not only for confirming the presence of disease but also to rule out the disease in non-CD subjects.

## 2. Materials and Methods

### 2.1. Patients and Sample Preparation

A total of 62 patients with suspected CD referred to the Pediatric Gastroenterology Unit of “G. Martino” University Hospital in Messina, Italy, between May 2020 and December 2020 were included in the study. The study protocol was approved by the Ethics Committee (Prot. Number 229–2020, date of approval: 11 November 2020). All patients with clinical suspicion of CD were evaluated by determining TGA-IgA in serum. Their demographic and clinical data are shown in Table 1. A whole blood sample was collected into 5 mL vacuum tubes and then separated by centrifugation at 3000 rpm for 10 min at room temperature. 2 mL of supernatant (serum) was used to perform an Enzyme-Linked ImmunoSorbent Assay (ELISA) test (Eurospital—Eu-tTG IgA, Eurospital Spa via Flavia 122, 34147, Trieste, Italy) for the detection of TGA-IgA; 1 mL of serum was stored at −80 °C to perform subsequent Raman analyses. In patients with a TGA-IgA value > 3 × ULN but ≤ 10 × ULN, 4 biopsies from the distal duodenum and at least one from the bulb were taken. A Marsh 2–3 type histological change was considered necessary to confirm the diagnosis of CD. Patients whose TGA-IgA values tested normal were considered the non-CD control group. IgA deficiency was ruled out by determining serum total IgA concentration. No patient with normal TGA-IgA titers resulted to have an IgA deficiency (defined as an IgA serum level below 7 mg/dL).

In the non-CD group, all other possible causes of intestinal inflammation were excluded by means of non-invasive or invasive tests. When clinically recommended, an upper gastrointestinal endoscopy was performed, and duodenal biopsies were taken in order to rule out a CD diagnosis. Finally, the non-CD group was composed of controls mainly with functional disorders in which CD or other inflammatory gastrointestinal diseases were ruled out.

Pediatric patients with TGA-IgA levels above 10 × ULN who fulfilled ESPGHAN guidelines for diagnosis without duodenal biopsy were excluded from final analysis. Patients with inconclusive diagnosis (i.e., potential CD) and with IgA deficiency (evaluated by determining total IgA titers) were also excluded. Final analysis was available for 48 patients, with 21 in the CD-group and 27 in the non-CD group.

ELISA TGA-IgA was measured with a commercial kit (Eurospital—Eu-tTG IgA, Eurospital Spa via Flavia 122, 34147, Trieste, Italy) according to the manufacturer instructions.

### 2.2. FT Raman Spectroscopy, Data Collection and Analysis

FT Raman serum analysis was performed using a DXR-SmartRaman Spectrometer (Thermo Fisher Scientific, Waltham, Massachusetts, USA). The DXR SmartRaman uses a diode laser (excitation wavelength: 785 nm). All Raman spectra were acquired over the wavenumber range of 3100–400 cm^−1^ (resolution: 1.9285 cm^−1^).

After accommodating the vials containing serum into their sample holder, the serum samples were analyzed by using the 180 Degree Sampling Accessory (Thermo Fisher Scientific, Waltham, MA, USA) and they were irradiated with a laser power of 24 mW emitting from a 50 µm circular spot. To increase signal to noise ratio (S/R), each Raman spectrum was the result of 32 sample exposure collections (duration of each exposure: 60.0 s). Total acquisition time was 32 min for each spectrum. All the Raman spectra were stored in SPA format, and post processing analysis was performed using the Omnic for dispersive Raman 9.0 software.

From obtained spectra, three spectral ranges were considered for evaluation: the 1750–1550 cm^−1^ spectral range, the 1500–1400 cm^−1^ and 1015–990 cm^−1^ ranges. From literature [9,38], the 1750–1550 cm^−1^ region corresponds to the Amide I band: it mainly consists of the C=O stretching vibration, with contributions from the C–N stretching and C–C–N deformation modes; the 1500–1400 cm^−1^ band is assigned to the CH_2_ and CH_3_ bending vibrations of protein; the last spectral range is assigned to the ring breathing mode of phenylalanine (Phe) [39]. To obtain diagnostic information from acquired spectra, the area ratio of the considered spectral ranges was calculated. In particular, the Phe ν-ring band located near 1003 cm^−1^ was used as an internal standard to normalize the spectra, as it has been reported to be insensitive to the micro-environment [40]. For this reason, the vibrational area bands between 1750 and 1550 cm^−1^ and between 1500 and 1400 cm^−1^ were normalized using the Phe area band centered at 1003 cm^−1^. The overall areas of the three spectral ranges used for diagnosis were obtained using the deconvolution function available on the Omnic for dispersive Raman 9.0 software. Deconvoluting the bands allowed for distinguishing between superimposed and very close bands and reducing band noise. For each spectral range, a Gaussian peak shape was chosen without any baseline correction. The spectral bands were analyzed by a curve-fitting procedure to evaluate the overall area of each band, and hereafter were indicated using their frequency centers, as A_1650_, A_1450_ and A_1003_. The A_1450_/A_1003_ and A_1650_/A_1003_ ratios have been computed.

### 2.3. Statistical Analysis

To evaluate the validity of Raman spectroscopy for the diagnosis of CD, sensitivity and specificity of the diagnostic test were determined by duodenal biopsy in the presence of positive celiac serology, as this is considered the GS for the diagnosis of CD. Receiver operating characteristic (ROC) curves were also determined. A ROC curve represents the plot of sensitivity vs 1-specificity [41]. In this study, sensitivity referred to the ability of Raman spectroscopy to correctly identify those patients with CD; conversely, specificity referred to the ability of Raman spectroscopy to correctly identify those patients without CD [42]. However, the relatively crude measures of sensitivity and specificity fail to take into account the cut-off point for a particular test, such in this case [43].

In fact, in clinical practice, if the cut-off changes, the frequencies of positive and negative results of the diagnostic test will vary [44]. The accuracy of any given threshold value can be measured by the probability of a true positive and the probability of a true negative result [45]. If the cut-off point is raised, the test is highly specific but not very sensitive. Similarly, if the cut-off point is low, the test is highly sensitive but not very specific. The area under the ROC curve (AUC) represents the overall accuracy of the diagnostic test. It utilizes values from 0 to 1, where a value of 0 indicates a perfectly inaccurate test and a value of 1 reflects a perfectly accurate test. In general, an AUC of 0.5 suggests no discrimination (i.e., ability to diagnose patients with and without the disease or condition based on the test), 0.7 to 0.8 is considered acceptable, 0.8 to 0.9 is considered excellent, and more than 0.9 is considered outstanding [46].

The optimal cut-off points were determined by using the Youden index. The Youden index, first introduced to the medical literature by Youden [47], is a function of sensitivity and specificity and is a commonly-used measure of overall diagnostic effectiveness. More importantly, the Youden index is the maximum vertical distance or difference between the ROC curve and the diagonal; it occurs at the cut-point that optimizes the biomarker’s differentiating ability when equal weight is given to sensitivity and specificity [48].

Sensitivity and specificity for the A_1450_/A_1003_ and A_1650_/A_1003_ ratios established cut-off points were calculated with their 95% confidence interval.

## 3. Results

**Figure 1:**
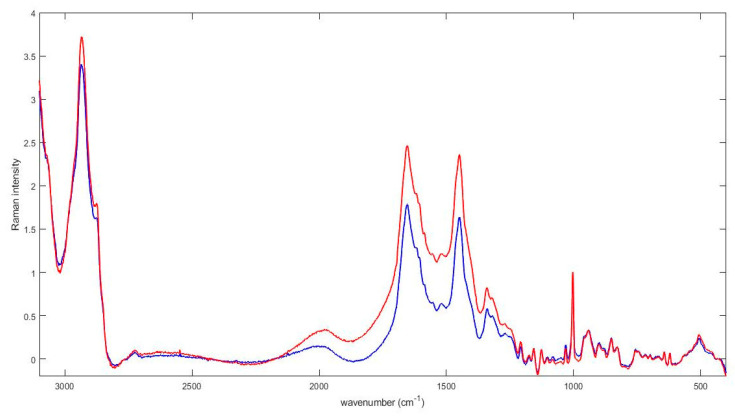
Average serum Raman spectra of non-CD (blue line) and CD (red line) patients. The three areas used for analysis were the 1550–1750 cm^−1^ spectral range, the 1400–1500 cm^−1^ spectral range, and the reference region of the Phe peak, around 1000 cm^−1^.

Serum Raman spectra were acquired from all samples successfully. In Figure 1 we report the average serum Raman spectra of non-CD and CD patients in the spectral range of 3100–400 cm^−1^.

Both spectra show the main typical protein vibrational modes, which derive from the polypeptide backbone (amide bands) and from aromatic and non-aromatic amino acid residue side chains. The tentative assignment of the main vibrational bands is reported in Table 2, taking into account the literature [32,48].

**Figure 2:**
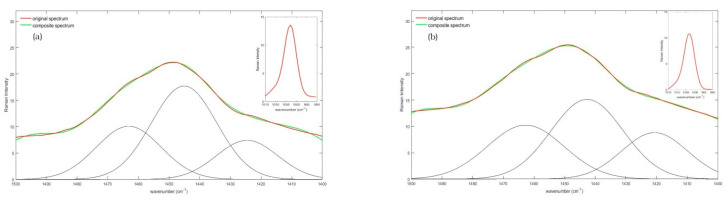
Average Raman spectrum, collected in the 1400–1500 cm^−1^ spectral range of serum from (**a**) non-CD and; (**b**) CD subjects, with the deconvoluted curves. In both spectra, the red line represents the original mean Raman spectrum, the black line identifies the Gaussian deconvoluted curves, and the green line shows the resulting composite spectrum, obtained from deconvolution computation. In the top right box of both figures the reference Phe peak is also displayed.

The acquired spectra, obtained from sera of non-CD and CD subjects, were visually similar, but a detailed analysis revealed that the areas of the investigated ranges were quite different. Our attention was focused on three ranges centered to ~1450 cm^−1^, ~1650 cm^−1^ and to ~1003 cm^−1^ respectively. In Figure 2 the 1500–1400 cm^−1^ spectral range is depicted. Figure 2a refers to the mean Raman spectrum of non-CD subjects, whereas Figure 2b shows the resultant mean spectrum of CD subjects.

**Figure 3:**
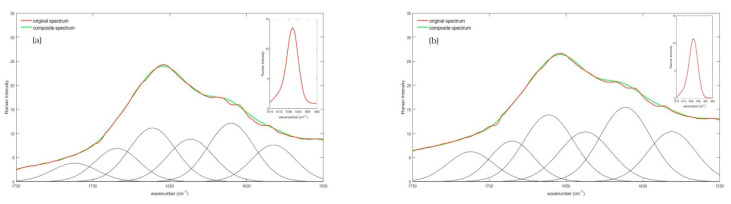
Average Raman spectrum, collected in the 1550–1750 cm^−1^ spectral range of serum from (**a**) non-CD and; (**b**) CD subjects. In both spectra, the red line represents the original mean Raman spectrum, the black line identifies the Gaussian deconvoluted curves, and the green line shows the resulting composite spectrum, obtained from deconvolution computation. In the top right box of both figures the reference Phe peak is also displayed.

Figure 3 shows mean Raman spectra of non-CD subjects (Figure 3a) and CD subjects (Figure 3b) acquired over the 1750–1550 cm^−1^ spectral range.

Differences in the average spectra of non-CD and CD subjects have been observed when the A_1450_/A_1003_ and A_1650_/A_1003_ ratios have been computed.

**Figure 4:**
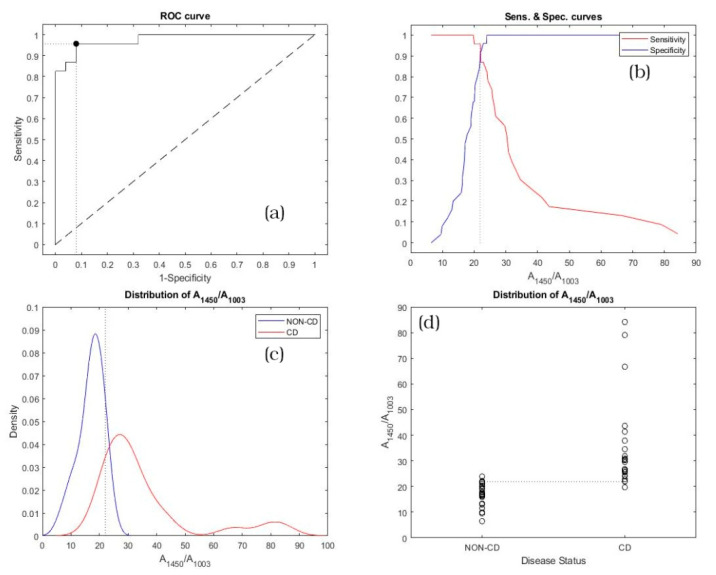
Statistical analysis related to the A_1450_/A_1003_ ratio for non-CD and CD subjects: (**a**) ROC curve; (**b**) sensitivity and specificity vs. A_1450_/A_1003_ ratios; (**c**) kernel distribution fit and (**d**) scatter distribution.

**Figure 5:**
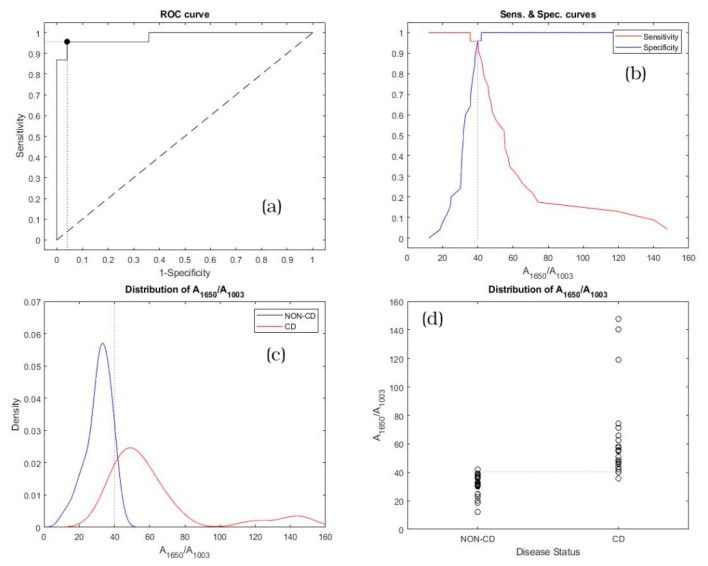
Statistical analysis related to the A_1650_/A_1003_ ratio for non-CD and CD subjects: (**a**) ROC curve; (**b**) sensitivity and specificity vs. A_1650_/A_1003_ ratios; (**c**) kernel distribution fit and (**d**) scatter distribution.

As a consequence, the A_1450_/A_1003_ and A_1650_/A_1003_ ratios have been calculated for all the subjects involved in the study, and the results of the statistical analysis are reported in Figure 4 and Figure 5.

Figure 4a plots the ROC curve for the A_1450_/A_1003_ ratio. In Figure 4a, the diagonal joining the point (0,0) and (1,1) is also represented (Line of equality) and the black point corresponds to the (1-Specificity, Sensitivity) calculated for the optimal cut-off for correctly identifying CD or non-CD subjects. Figure 4b shows the trend of sensitivity (red line) and specificity (blue line) vs. A_1450_/A_1003_ ratios. The vertical dot line identifies the optimal cut-off, and the point of intersection of sensitivity and specificity curves corresponds to the black marker depicted in Figure 4a. Figure 4c shows the kernel distribution fit for non-CD (blue line) and CD (red line) subjects across all A_1450_/A_1003_ ratios. Also, in this case the vertical dot line represents the optimal cut-off. Figure 4d displays the scatter distribution of each group (non-CD and CD subjects). In Figure 4d, the optimal cut-off is plotted as the horizontal dot line.

Figure 5a plots the ROC curve calculated for the A_1650_/A_1003_ ratio. The line of equality and the optimal cut-off point are also depicted. Figure 5b shows the trend of sensitivity (red line) and specificity (blue line) vs. A_1650_/A_1003_ ratios. Also, in this case the optimal cut-off is displayed as a vertical dot line. The kernel distribution fit for non-CD (blue line) and CD (red line) subjects is plotted in Figure 5c, whereas the scatter distribution of A_1650_/A_1003_ ratios vs. the disease status is displayed in Figure 5d. The optimal cut-off, displayed in Figure 5c,d, is represented as the vertical and the horizontal dot line, respectively. As previously reported, the optimal cut-off lines in Figure 5b–d correspond to the marker black point on Figure 5a.

To verify the overall accuracy of the diagnostic test, AUC values for both ratios have been calculated and the computations of AUC are reported in Table 3. The lower and the upper limits, which represent the 95% confidence interval, are also indicated in the same table.

The optimal cut-off points for both ratios, established following the Youden method, are reported in Table 4. In the same table, the corresponding sensitivity and specificity are also indicated, with the lower and upper limits at 95% confidence interval.

## 4. Discussion

In this study, Raman spectroscopy was used to analyze sera from patients that were referred for suspicion of CD. We focused our attention on three bands to compute the methodology used to discriminate non-CD patients from CD patients. The considered bands were Amide I, the scissoring deformation of the CH_2_ vibrational mode of Amide II and the Phe band at ~1003 cm^−1^, which is used as an internal standard to normalize the spectra. The Amide I band (centered at about 1650 cm^−1^) is most commonly used to interpret changes in the protein secondary structure. This is in part due to the overlay of the Amide II and Amide III bands with the vibrational frequencies of certain stretching modes, such as C–C, C–N and CH_2_, which substantially complicates their assignment and interpretation [52,53,54]. The band centered at ~1450 cm^−1^ is commonly used to determine the amount of protein structure, which can be attributed to unordered parts of amyloid fibrils. Consequently, it can be used to estimate the amount of hydrophobic fibril core [32]. These bands constitute an excellent marker to quantify the secondary structure and conformational changes of proteins as a consequence of the role played by the amide moiety in crosslinking [55]. We know that transglutaminase has a crucial role in CD. Transglutaminases (TG) belong to a family of structurally and functionally related enzymes that catalyse Ca^2+^-dependent post-translational modifications of proteins by introducing protein-protein cross-links, amine incorporation, and site-specific deamidation. In CD, tTG is a specific target of a conditional autoimmune mechanism driven by exogenous cereal peptides. In genetically predisposed individuals, ingestion of wheat, rye and barley leads to small intestinal villous atrophy, malabsorption, and the production of antibodies against tTG. Gluten peptides derived from these cereals are rich in glutamine and proline residues (especially those from the alcohol-soluble gliadin fraction of gluten) and are good substrates for the transamidating enzyme reaction catalyzed by tTG. Given the crucial role of transglutaminase in CD, it is not surprising that the spectrum of CD patients differs from that of controls in specific regions involving conformational modification of proteins concerned in transamidation and deamidation by transglutaminase. tTG is expressed in small intestine, and that expression is increased in untreated celiac small intestinal mucosa, where tTG is detected at the level of muscularis mucosae and pericryptal fibroblasts adjacent to enterocytes [56]. Moreover, cell surface tTG was found on macrophages and dendritic cells, which are known to play an important role in the pathogenesis of CD. tTG expression was detected in celiac enterocytes, together with an evident upregulation of the enzymatic activity in the subepithelial layer. More than 25 endogenous proteins by a proteomic approach in an enterocyte-like system were identified, both acyl-acceptor and acyl-donor, that might represent putative substrates for tTG and, thus, potential neoantigens recognized by the immune system [57]. All this evidence suggests an active role of tTG inside and outside the small intestine in protein conformational changes in CD patients. In this context, the analysis of the spectra obtained from CD and non-CD subjects’ sera ones proves that Raman spectroscopy is able to distinguish between the samples furnishing a rapid, non-invasive diagnostic method of CD. In fact, observing the obtained results of Table 2, the AUC values lie between 0.97 and 1.0; therefore, the test can be considered outstanding and both the A_1450_/A_1003_ and A_1650_/A_1003_ ratios represent excellent markers in CD diagnosis. Considering Table 3, the obtained sensitivity values were 0.957 with a 95% confidence interval (95% CI) of 0.781–0.999, regarding both the A_1450_/A_1003_ and A_1650_/A_1003_ markers. The obtained specificity values were 0.920 with a 95% CI of 0.740–0.990 and 0.960, CI 95%, of 0.796–0.999, regarding the A_1450_/A_1003_ and A_1650_/A_1003_ markers, respectively.

Therefore, a subject is assessed as affected (positive) if the A_1450_/A_1003_ and A_1650_/A_1003_ tested marker values are greater than the 21.97 and 40.14 threshold values, respectively; otherwise, the subject is diagnosed as a non-CD subject.

## 5. Conclusions

The GS for the diagnosis of CD is represented by duodenal biopsy, which is resource intensive, time-consuming and invasive for the patient. Non-invasive methods to rule out CD diagnosis have been proposed; in pediatric patients, the high specificity and sensitivity of tTG in the context of high pre-test probability [5] allows clinicians to avoid duodenal biopsy to confirm CD. In our study, Raman spectroscopy analysis shows a diagnostic accuracy comparable with that of anti-tTG. If confirmed prospectively in a wider cohort of patients, Raman spectroscopy analysis may be considered as a suitable alternative to classic CD diagnostic tools. Noteably, Raman spectroscopy has the advantage of being relatively cheap, time-sparing, and safe.

## 6. Patents

The developed methodology is patented (Patent No. 102019000007214–06.04.2021).

## Figures and Tables

**Table 1:** Demographic and baseline features in CD and non-CD patients.

	CD	Non-CD
Median age and SD ^1^	15.6 (3.15)	12.6 (5.17)
M:F ratio	1.4	1.5
Final diagnosis in non-CD group	-	IBS * (50%), RAP ** (30%), Functional dyspepsia ^#^ (15%), Iron deficiency Anemia (5%)

^1^ Standard deviation; * irritable bowel syndrome; ** recurrent abdominal pain; ^#^ in these cases duodenal biopsies were performed in order to rule out CD diagnosis.

**Table 2:** Tentative assignment of the main vibrational bands obtained from sera analysis.

Center Frequency (cm^−1^)	Tentative Assignment	References
520	Disulfide band	[32]
759	Ring vibration of tryptophan	[33]
830 and 850	Tyrosine doublet	[33,34]
1003	Phenylalanine	[40]
1300 band	Amide III vibration	[49]
1450 band	CH_2_ scissoring deformation	[49,50]
1550	Amide II vibration	[39]
1650	Amide I vibration	[38]
2935	C–H Stretching vibration	[51]

**Table 3:** AUC results, lower and upper limits at 95% confidence interval, related to A_1450_/A_1003_ and A_1650_/A_1003_ ratios.

Marker	AUC	Lower Limit	Upper Limit
A_1450_/A_1003_	0.97739	0.85208	0.99984
A_1650_/A_1003_	0.98087	0.85817	0.99994

**Table 4:** Optimal cut-off points obtained following the Youden method, sensitivity, and specificity (values, lower and upper limits at 95% confidence interval), related to A_1450_/A_1003_ and A_1650_/A_1003_ ratios.

Marker	Cut-Off Point	Sensitivity	Specificity
A_1450_/A_1003_	21.97	0.957 (LL ^1^: 0.781; UL ^2^: 0.999)	0.920 (LL ^1^: 0.740; UL ^2^: 0.990)
A_1650_/A_1003_	40.14	0.957 (LL ^1^: 0.781; UL ^2^: 0.999)	0.960 (LL ^1^: 0.796; UL ^2^: 0.999)

^1^ Lower Limit; ^2^ Upper Limit.
